# A Comparison of Facial Color Pattern and Gazing Behavior in Canid Species Suggests Gaze Communication in Gray Wolves (*Canis lupus*)

**DOI:** 10.1371/journal.pone.0098217

**Published:** 2014-06-11

**Authors:** Sayoko Ueda, Gaku Kumagai, Yusuke Otaki, Shinya Yamaguchi, Shiro Kohshima

**Affiliations:** 1 Tokyo Institute of Technology, Tokyo, Japan; 2 Tama Zoological Park, Tokyo Zoological Park Society, Tokyo, Japan; 3 Yokohama Zoological Gardens, Yokohama, Japan; 4 Wildlife Research Center of Kyoto University, Kyoto, Japan; Institut Pluridisciplinaire Hubert Curien, France

## Abstract

As facial color pattern around the eyes has been suggested to serve various adaptive functions related to the gaze signal, we compared the patterns among 25 canid species, focusing on the gaze signal, to estimate the function of facial color pattern in these species. The facial color patterns of the studied species could be categorized into the following three types based on contrast indices relating to the gaze signal: A-type (both pupil position in the eye outline and eye position in the face are clear), B-type (only the eye position is clear), and C-type (both the pupil and eye position are unclear). A-type faces with light-colored irises were observed in most studied species of the wolf-like clade and some of the red fox-like clade. A-type faces tended to be observed in species living in family groups all year-round, whereas B-type faces tended to be seen in solo/pair-living species. The duration of gazing behavior during which the facial gaze-signal is displayed to the other individual was longest in gray wolves with typical A-type faces, of intermediate length in fennec foxes with typical B-type faces, and shortest in bush dogs with typical C-type faces. These results suggest that the facial color pattern of canid species is related to their gaze communication and that canids with A-type faces, especially gray wolves, use the gaze signal in conspecific communication.

## Introduction

The facial morphology and color pattern of animals, especially those around the eyes, have been suggested to serve various adaptive functions related to the gaze signal that facilitate the detection of their gaze direction by other animals. For example, Cott [1] pointed out that various predators camouflage their eyes to increase their hunting success. Kobayashi and Kohshima [2], [3] noted that the morphology of human eyes, with a horizontally elongated outline and a large area of exposed white sclera lacking pigmentation, is unique among primates. They discussed that humans have “gaze-signaling eyes” that enhance the gaze signal for their developed gaze-communication, whereas most other primates have “gaze-camouflaging eyes” that camouflage the gaze signal by pigmented sclera.

Similar to humans, gray wolves (*Canis lupus*) have facial color patterns in which the gaze direction can be easily identified, although this is often not the case in other canid species. For example, identifying the gaze direction of raccoon dogs (*Nyctereutes procyonoides*) appears to be difficult because of the dark facial color around their eyes. Various canid species reportedly use body postures and facial expressions in their visual communication [4], and domestic dogs and gray wolves can follow the gaze direction of human and conspecifics [5]–[7]. In canid species, however, no comparative studies of facial color patterns, with particular focus on the gaze signal, have been conducted to date.

In the present study, we compared facial color patterns among 25 canid species, focusing on the gaze signal. We analyzed the relationship between facial color pattern and phylogeny, sociality, and hunting behavior to estimate the function of facial color patterns in canid species. In particular, we tested the hypothesis that gaze-signaling facial color patterns similar to those of the gray wolf are used for visual communication among conspecifics using the gaze. In this case, we expected that species with gaze-signaling faces would tend to engage in group-living and/or group-hunting, whose communication needs might be larger than for solo/pair-living and/or solo-hunting canids. To compare the intensity of the gaze signal to conspecifics, we measured the color contrast among five facial parts around the eyes, assuming that the color vision of canid species closely resembles that of human deuteranopia [8], [9]. We also compared the gazing behaviors of three species with different types of facial color pattern: gray wolves with the most intense gaze-signaling faces, in which both pupil position in the eye outline and eye position in the face are clear; fennec foxes with intermediate gaze-signaling faces, in which only the eye position is clear; and bush dogs with a typical gaze-camouflaging face, in which both the pupil and eye position are unclear. All three species engage in group living, forming family groups of more than three individuals even during the non-breeding season. If the facial color pattern is related to gaze-signal communication, species with gaze-signaling faces are expected to exhibit longer-duration gazing behaviors that display their gaze signal to others compared to gaze-camouflaging species. Fox [4] compared the behaviors of many canid species and documented that direct staring at other individuals and behaviors that avoid the gaze of other individuals occur in various canid species including gray wolves. To discuss whether another possible adaptive function of iris color (protection against ultraviolet radiation) can explain the light colored irises of gray wolves, an important aspect of the gaze-signaling face, we also compared a lightness index of iris color among subspecies of wolves living in arctic, temperate, and subtropical areas.

Our results suggested that the facial color patterns of canid species are related to their gaze communication and that gray wolves use the gaze signal in conspecific communication. Thus, the present study provides a novel perspective for future research on the morphology and communication of canid species.

## Materials and Methods

### Facial Images of Canid Species

Facial images without color manufacturing for 320 adult individuals of 25 species (1–127 individuals per species, mean  = 13) were collected (Table S1). Facial images of 24 species were collected from published photographs, books, and Web sites. We also photographed the facial images of 14 species using a digital camera (Canon EOS Kiss x and Nikon D80) at zoos.

### Analysis of Facial Images

**Figure 1:**
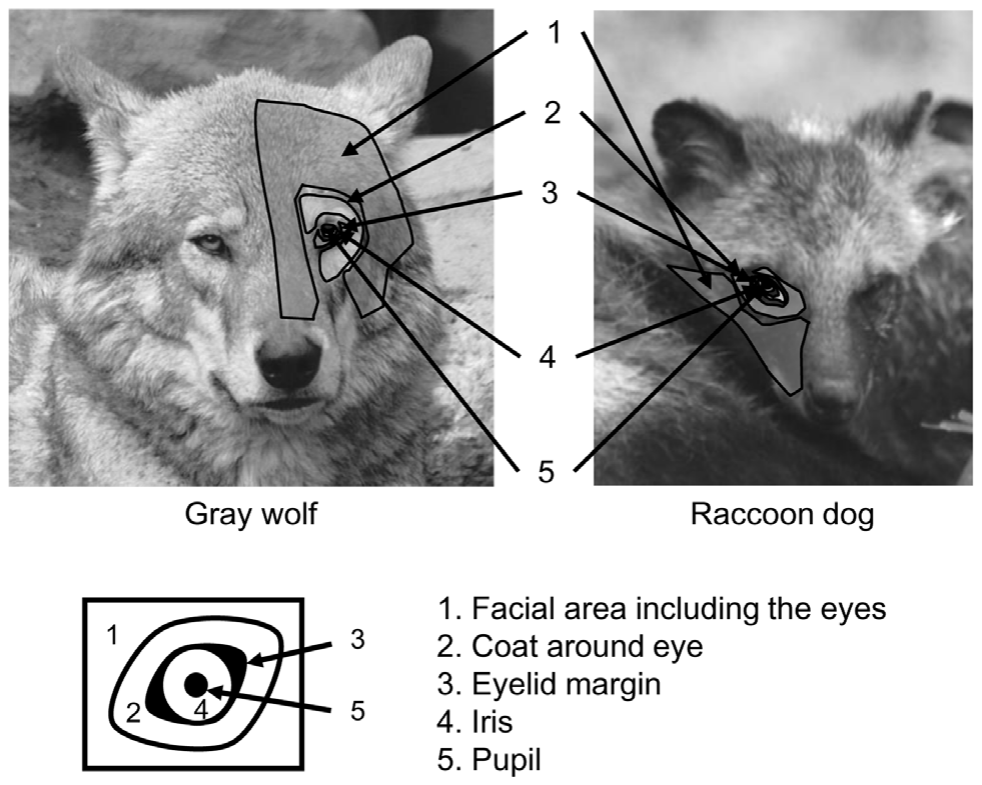
Analyzed parts of the face. All studied canid species had a dark-colored eyelid margin.

Using these facial images, color contrasts between the following five parts of the face were analyzed as indices of the gaze signal: pupil (P), iris (I), eyelid margin (E), coat around the eyes (C), and facial area including the eyes (F) (Figure 1). All studied species had a dark-colored eyelid margin.

The color contrasts were calculated assuming that the color vision of canid species closely resembles that of human deuteranopia [8], [9]. In this analysis, we measured RGB color values of each part and converted these into Lab-color values of human deuteranopia (i.e., removing green color effects) according to the methods of Viénot et al. [10]. We then measured.0 the luminance of each facial part in this Lab-color space. RGB measurements were conducted using the computer software Photoshop 7.0 (Adobe).

Using these luminance values, Michelson contrasts between the following parts were calculated using the following formula,
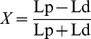
where Lp is the luminance of pale color parts and Ld is the luminance of dark color parts.

Here, we used the following four color contrast values as indices of the gaze signal: 1) contrast between the iris and pupil (IP) as an index for the conspicuousness of pupil position in the eye outline, 2) the iris–eyelid margin contrast (IE), 3) the coat around the eye–eyelid margin contrast (CE), and 4) the coat around the eye–face coat contrast (CF) as indices for the conspicuousness of eye position on the face.

### Phylogenic Analysis

In the analysis of the relationship between facial color pattern and phylogeny, we referred to the phylogeny of canid species estimated by Lindblad-Toh et al. [11]. They analyzed nuclear exons and introns of 30 of 34 living canid species and categorized them into the following four clades: red fox-like clade (10 species), South American clade (four species), wolf-like clade (10 species), and the gray and island fox clade (one species). Three study species that were not examined in their report were categorized as follows: Bengal foxes into the red fox-like clade and dingoes and red wolves into the wolf-like clade.

### Definition of Sociality of the Studied Canid Species

To analyze the relationship between facial color pattern and sociality in the studied canid species, we categorized the species into the following two types based on their social life in the non-breeding season: the group-living type that forms a family group of more than three individuals even during the non-breeding season and the solo/pair-living type that never forms groups larger than a pair (Table S1). We also categorized the study species into the following two types based on their hunting behavior: the group-hunting type that hunts in groups of more than three individuals and the solo-hunting type that hunts alone or in pairs (Table S1). These social-life and hunting behaviors were categorized with reference to previous reports [12]–[15]. In the present study, we regarded the kit fox (*Vulpes macrotis*) and swift fox (*Vulpes velox*) as the same species. Because gray wolves, including the high arctic wolf, red fox, and arctic fox exhibit different body color types, we analyzed the facial color pattern of each type separately.

### Observation of Gazing Behavior

We compared the gazing behaviors of the following three species that engage in group living: gray wolves (*C. lupus*), fennec foxes (*Vulpes zerda*) and bush dogs (*Speothos venaticus*) under captive conditions. Gazing behavior was defined as a behavior during which the animal fixes its face direction toward the other individual for over 1 s, keeping its body still. We also compared “face-averting” behavior in which the animal kept its face averted from the other animal in the vicinity of that animal for over 1 s. We observed these behaviors in 11 gray wolves from the same family group (five adult males and six adult females) at the Tama Zoological Park, in a pair of fennec foxes (one adult male and one adult female) at the Kyoto City Zoo, and in four bush dogs (one adult male and three adult females) from two family groups kept in two separate groups (a group of three males and a group of three females) in the Yokohama Zoological Gardens in Japan. Each gray wolf was observed for an average of 2.60 h (1.70–2.88 h) during the daytime (09:00–13:00) on 9 March 2011. Each of the two fennec foxes was observed for an average of 3.23 h (2.98 and 3.48 h, respectively) during the daytime (09:00–13:00) on 6 and 7 February 2014. Each bush dog was observed for an average of 2.56 h (2.41–2.66 h) during the daytime (09:30–13:00) on 12 October 2011 and in the afternoon (13:30–16:30) on 13 October 2011. All observations were conducted during the breeding season of each species [16]–[18] when interactions among the group members were considered to occur more frequently than during the non-breeding season.

### Ethical Statement

Behavioral observations during this study were performed with official permission from the Tama Zoological Park, the Kyoto City Zoo, and the Yokohama Zoological Gardens. All images of animals used in this study were taken at zoos, other private facilities, and in their natural habitats with official permission or at locations where no specific permission was required for photography and video recording. This study was conducted in strict accordance with the recommendations in the guidelines for the treatment of animals in behavioral research and teaching of the Association for the Study of Animal Behaviour. No invasive methods were used in this study. All images used in this study were photographed or video-recorded during the observation of free-ranging animals under captive or wild conditions without the use of a strobe light.

### Statistical Analyses

Mann-Whitney *U*-tests, Kruskal-Wallis tests, Fisher's exact tests, and Tukey WSD tests were used for comparisons of facial color patterns. Kruskal-Wallis tests and Steel-Dwass tests were used for comparisons of gazing behaviors.

## Results

**Figure 2:**
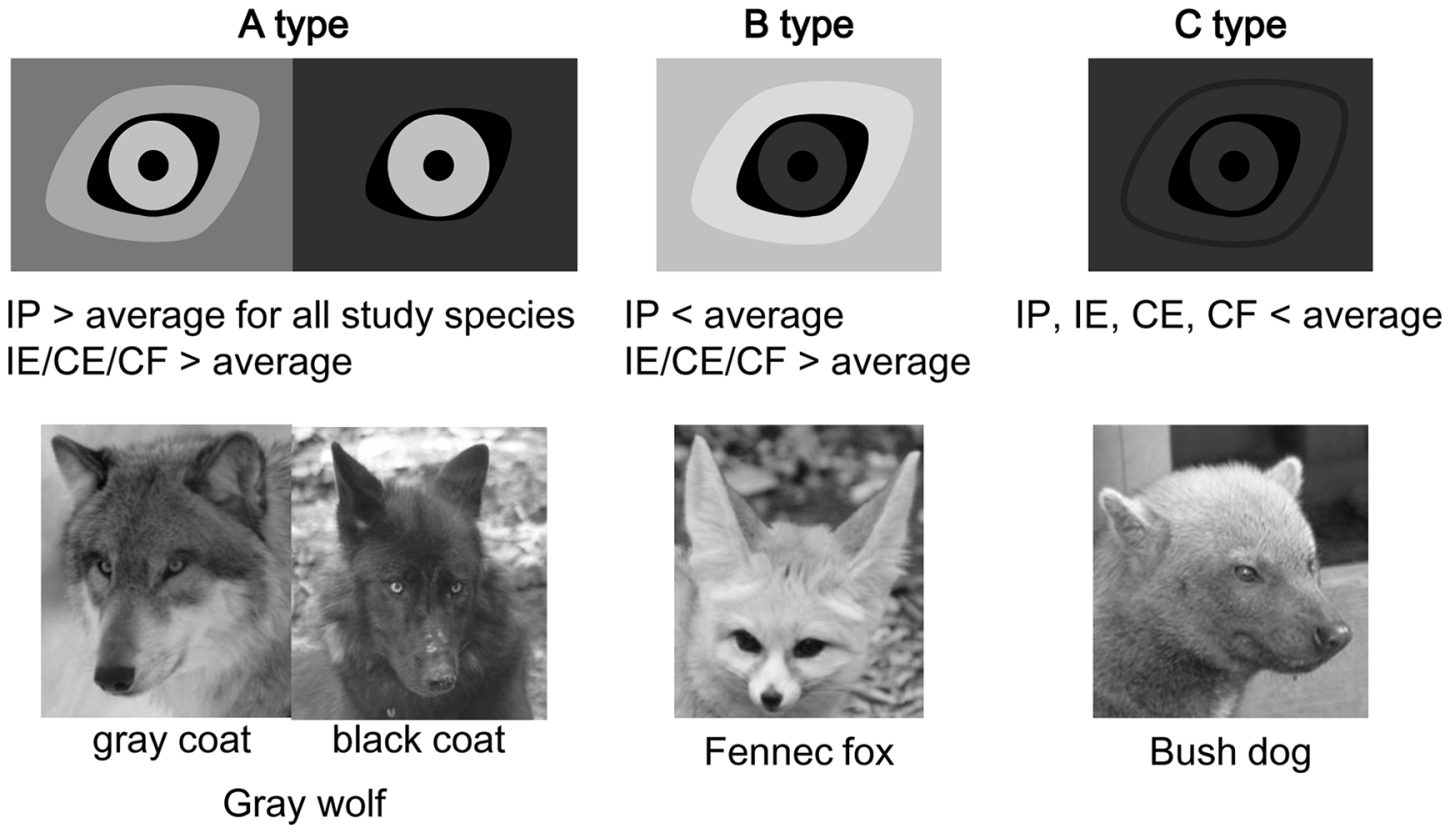
Three types of facial color pattern. Images of gray wolf, fennec fox, and bush dog showed typical A-, B-, and C- type faces, respectively: in A-type, both the pupil position in the eye outline and the eye position in the face are conspicuous; in the B-type, only the eye position is conspicuous; and in C-type, both the pupil and eye position are inconspicuous.

**Figure 3:**
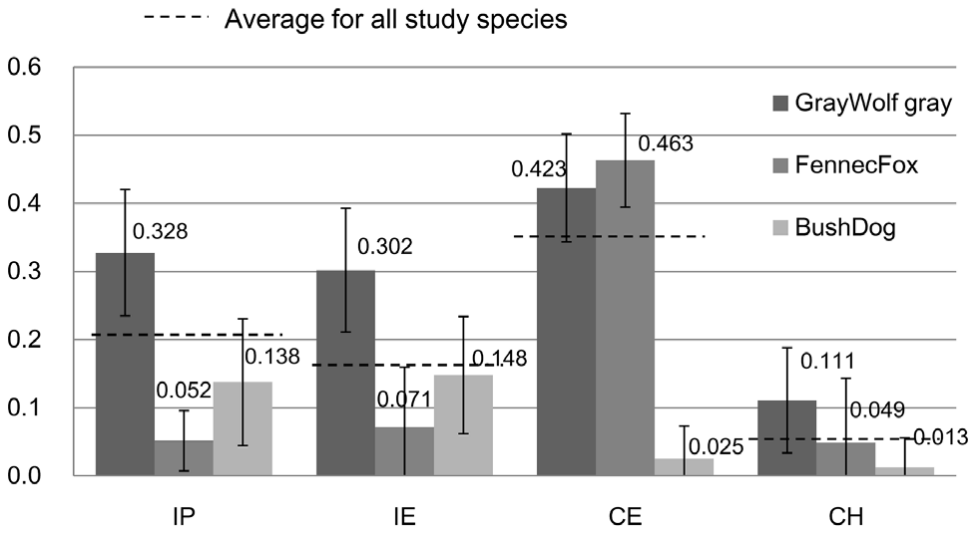
Contrast indices of the three focal species. Mean values of the four contrast indices of the three focal species (gray wolves, fennec foxes, and bush dogs). Vertical bars indicate the standard deviation. Dotted lines show the mean values of indices among all studied canid species. Iris – Pupil contrast (IP) means the conspicuousness of the pupil position in the eye outline. Iris – Eyelid margin contrast (IE), Coat around eyes – Eyelid margin contrast (CE), and Coat around eyes – Facial coat contrast (CF) mean the conspicuousness of the eye position on the face.

The facial color patterns of the study animals could be categorized into the following three types based on the four contrast indices (Figures 2 and 3, Table S2): the A-type, in which both the pupil position in the eye outline and the eye position in the face were conspicuous (IP > average for all study species, and IE or CE or CF > average); the B-type, in which only the eye position on the face was conspicuous (IP < average, and IE or CE or CF > average); and the C-type, in which both the pupil and eye position were inconspicuous (IP, IE, CE, and CF < average). The A, B, and C-type included 11, 9, and 6 species, respectively.

### Relationship between Facial Color Type and Phylogeny

**Figure 4:**
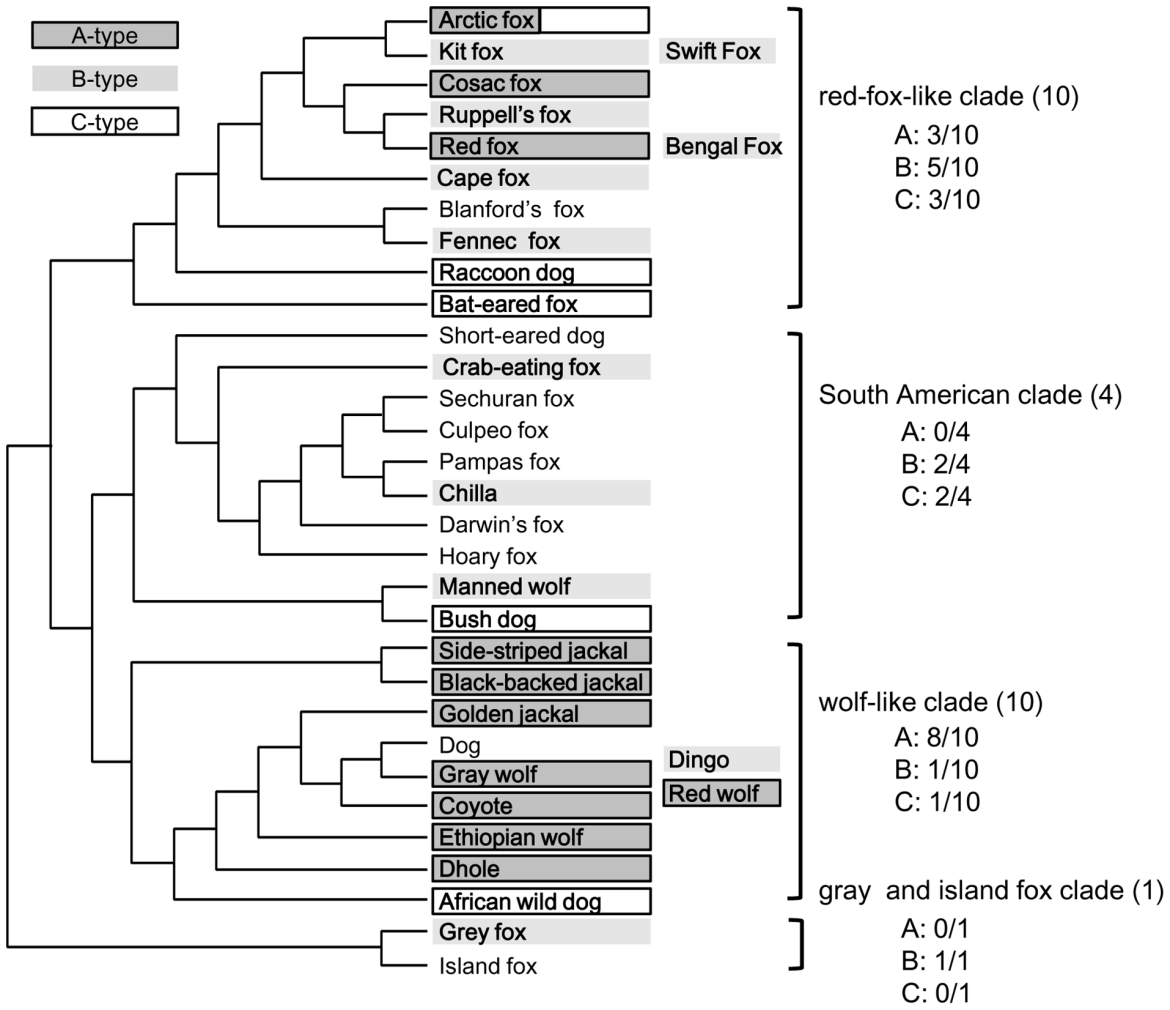
Relationship between the facial color type and phylogeny of canid species. The phylogenic tree is based on [11]. Three study species that were not examined in their report were categorized as follows: Bengal foxes into the red fox-like clade and dingoes and red wolves into the wolf-like clade. The proportion of facial color types differed significantly across clades of canid species (p = 0.030).

The proportion of facial color types differed significantly across clades of canid species (Fisher's exact test, p = 0.030; Figure 4). Species with A-type faces were only observed in the red fox-like clade (3/10 species) and in the wolf-like clade (8/10 species). In contrast, species with B-type faces were observed in all four clades, and those with C-type faces were observed in three clades.

### Relationship between Facial Color Type and Sociality

**Figure 5:**
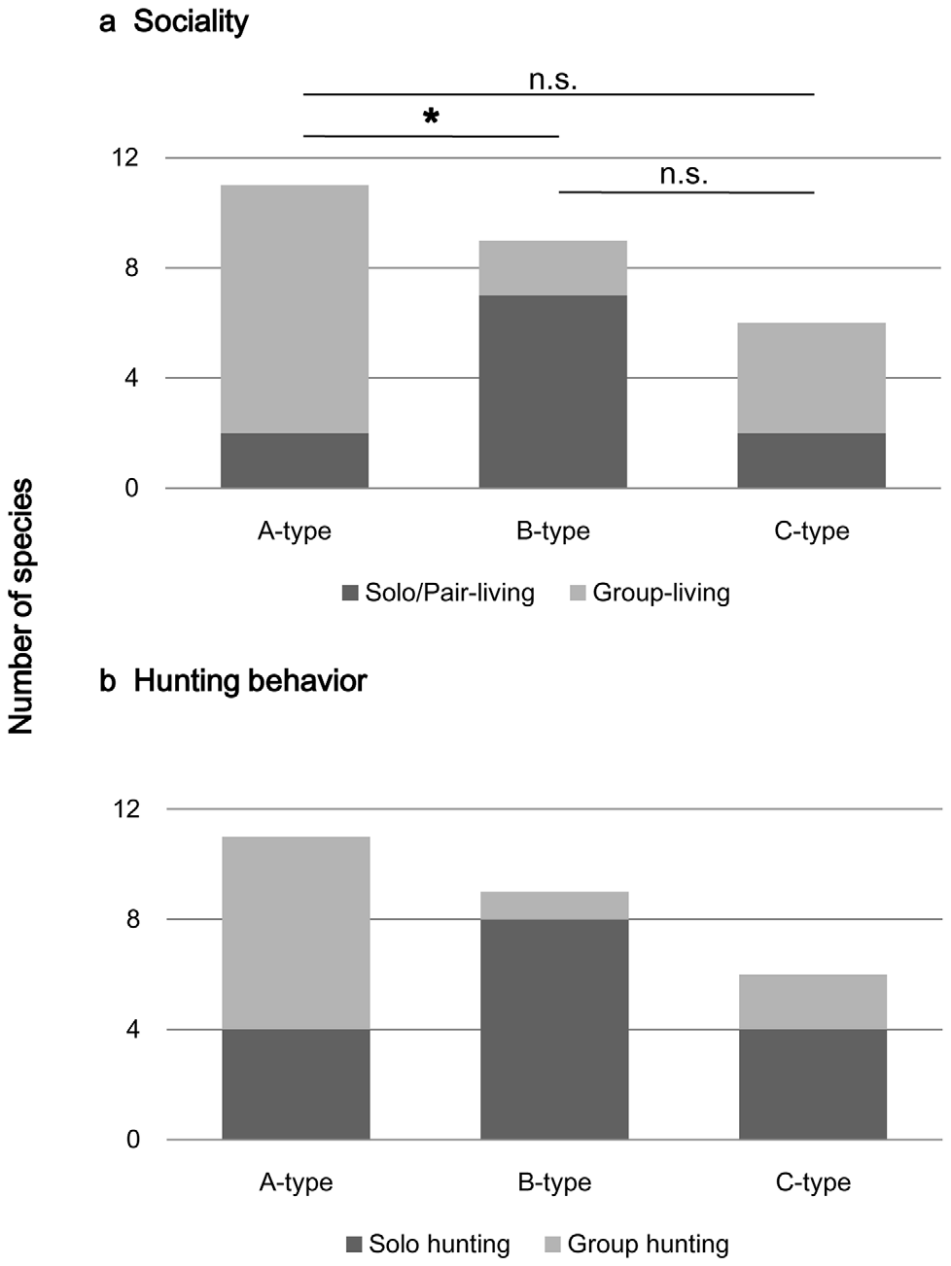
Relationship between facial color types and sociality and hunting behavior of the studied canid species. a) The proportion of facial color types differed significantly based on the sociality (p = 0.022), and the proportion of social types differed significantly between species with A-type faces and those with B-type faces (*: p<0.05). b) The proportion of facial color types differed marginally significant between the two types of hunting behaviors (p = 0.056).

**Figure 6:**
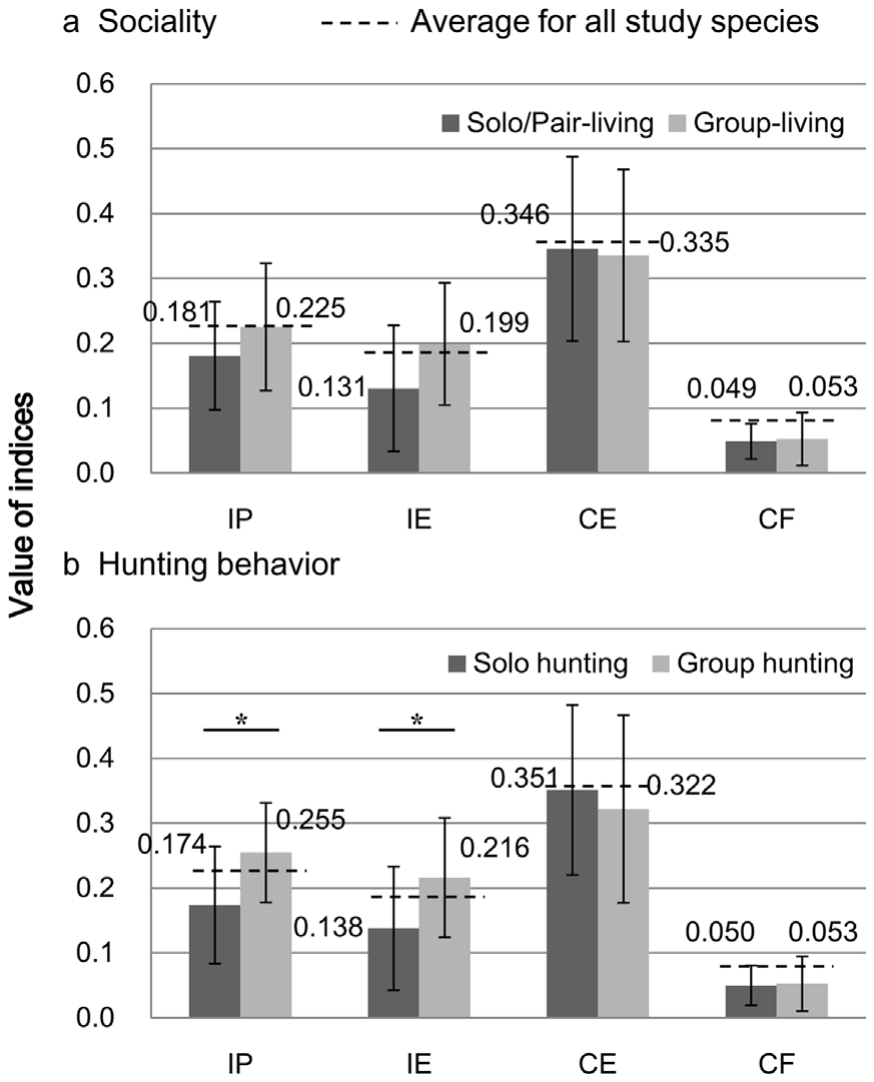
Difference of the four contrast indices on average in sociality and in hunting behavior of the studied canid species. Vertical bars indicate the standard deviation. Dotted lines indicate the average of the indices among all studied canid species. The four contrast indices did not significantly differ between group-living and solo/pair-living species. The contrast indices reflecting the lightness of iris color (IP and IE) of the group-hunting species were significantly higher than those of solo-hunting species (p = 0.029 for IP and p = 0.043 for IE, *: p<0.05).

The proportion of facial color types differed significantly based on the sociality of the canids during the non-breeding season (Fisher's exact test, p = 0.022; Figure 5a). The proportion of social types differed significantly between species with A-type faces and those with B-type faces (Tukey WSD, p<0.05). Species with A-type faces included more group-living species than solo/pair-living species. In contrast, species with B-type faces included more solo/pair-living species. The four contrast indices did not significantly differ between group-living and solo/pair-living species (Mann-Whitney *U*-test, p = 0.219, 0.067, 0.451, and 0.936 for IP, IE, CE, and CF, respectively; Figure 6a).

### Relationship between Facial Color Type and Hunting Behavior

The proportion of facial color types differed marginally significant between the two types of hunting behaviors (Fisher's exact test, p = 0.056; Figure 5b); the proportion of group-hunting species with A-type faces was higher than for species with other face types. In addition, the proportion of solo-hunting species with B-type faces was higher than for species with other face types. The contrast indices reflecting the lightness of iris color (IP and IE) of the group-hunting species were significantly higher than those of solo-hunting species (Mann-Whitney *U*-test, p = 0.029 for IP and p = 0.043 for IE; Figure 6b), although no significant differences were observed for CE or CF (Mann-Whitney *U*-test, p = 0.274 for CE and 0.903 for CF).

### Gazing Behavior of the Three Group – Living Species

Gazing behavior was observed in three focal species. Gray wolves performed gazing behaviors in a wider variety of postures (six postures) compared to fennec foxes (three postures) or bush dogs (three postures). In particular, the pointing posture was not observed in either fennec foxes or bush dogs. The pointing posture is a characteristic behavior of gray wolves and domestic dogs in which the animal fixes its face and body toward the other animal, assuming the posture when preparing to dash or pounce, often keeping its foreleg up [19]. Although pointing did not occur as frequency as the other postures, the duration time (7.67 s) and maximum duration time (28.0 s) were very long.

**Figure 7:**
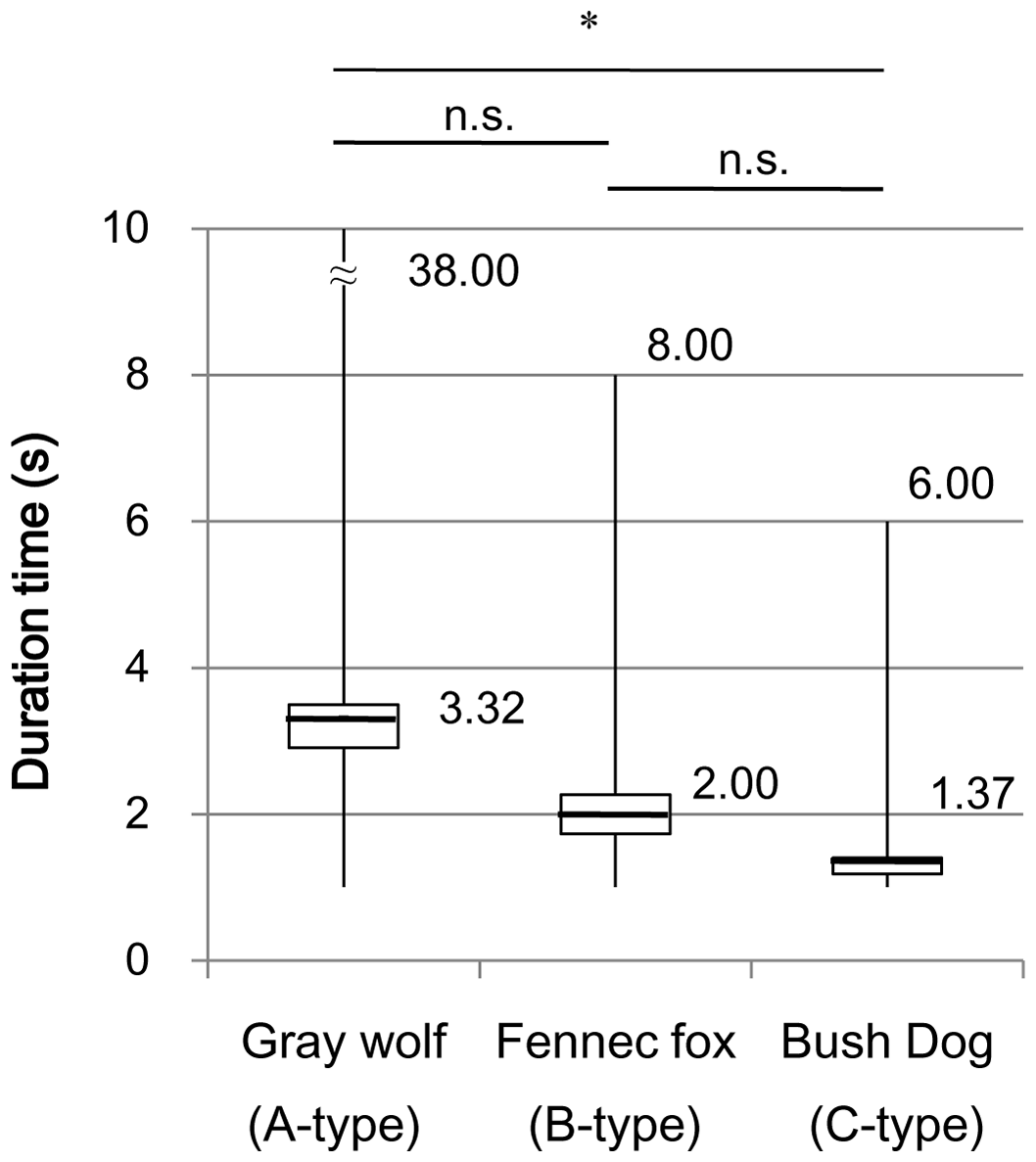
Duration time of gazing behaviors in the three focal species. The mean duration time of the gazing behaviors differed significantly among the three study species (gray wolves, fennec foxes, and bush dogs, p = 0.005). The mean duration time of gazing behaviors was significantly longer in gray wolves than in bush dogs (p = 0.011, *: p<0.05).

The mean duration time of the gazing behaviors significantly differed among the three study species (Kruskal-Wallis test, p = 0.005; Table S3). Gazing behaviors lasted the longest in gray wolves with typical A-type faces, occurred for intermediate length of time in fennec foxes with typical B-type faces, and occurred for the shortest durations in bush dogs with typical C-type faces. The mean duration time of gazing behaviors was significantly longer in gray wolves than in bush dogs (Steel-Dwass test, p = 0.011; Figure 7). The maximum duration times of gazing behaviors in gray wolves (38.0 s) were also much longer than those of fennec foxes (8.00 s) and bush dogs (6.00 s). In contrast, no significant differences were observed in the frequency of gazing behaviors per target individual within the observed species (Kruskal-Wallis test, p = 0.377; Table S3). Face-averting behaviors, however, were observed in all three species. Neither the duration time nor frequency of face-averting behaviors significantly differed across species (Kruskal-Wallis test, p = 0.859 for duration time; P = 0.137 for frequency).

### Relationship between Iris Color and Distribution Area among Wolf Subspecies

Lightness of iris color (IP, the most important aspect of the gaze-signal in the A-type faces) did not differ significantly among high arctic wolves (IP  = 0.207, n = 3), Canadian wolves (IP  = 0.357, n = 16), or Mexican wolves (IP  = 0.269, n = 9) living in arctic, temperate, and subtropical areas, respectively (Kruskal-Wallis test, p = 0.135).

## Discussion

Light-colored irises in A-type faces are the most important aspect of the gaze-signal in the facial color pattern of canid species. This feature results in high contrast between the iris and the dark color of the pupil (IP), making the pupil position conspicuous within the eye outline. Because most parts of the eye outline are covered by the iris and the area of exposed sclera is very small in all canid species, light-colored irises enhance the gaze signal, just as the white sclera of humans highlights the position of dark-colored irises within the eye outline. In addition, because the eyelid margin was dark-colored in all of the study canid species, light-colored irises also strongly contrast with the eyelid margin (IE), making the eye position conspicuous on the face. In contrast, the dark-colored irises of B- and C-type faces result in low contrast with the pupil and eyelid margin, thus reducing the gaze signal.

Facial color patterns change with growth in many canid species, although no studies have directly examined such developmental changes. For example, all newborn gray wolves observed in the present study had dark-colored bodies and C-type faces with dark-colored irises. Their body color became lighter after 2 weeks, and their facial color patterns changed from C-types to B-types before reaching about 6 months old and to A-types with light-colored irises before reaching 1 year old (Ueda et al., unpublished data). Some previously published images [20]–[23] also support these observations. Obee [21] also stated that gray wolf cubs had “the blue eyes of infancy.” Several other canid species, such as dingoes and swift foxes, also appear to change facial color patterns from C-type to B-type with growth (from images in [12], [24]), whereas some species, such as bush dogs, never change their facial color type or iris color (observations at the Kyoto City Zoo and the Nagoya Higashiyama Zoo).

Although the physiological basis of eye color in canid species has not yet been examined directly, the iris color as well as the skin and hair color of humans and other mammals are reportedly pigmented by two kinds of melanin, eumelanine (black/brown color) and pheomelanin (red/yellow color), in melanocyte cells [25], [26]. Therefore, the light-colored (bright yellow) irises of A-type faces of gray wolves and the dark-colored irises of B- and C-type faces are likely due to pheomelanin and eumelanine, respectively. Changes in iris color with growth may also be caused by changes in the proportions and amounts of these two types of melanin.

One possible adaptive function of iris pigmentation is protection against ultraviolet (UV) radiation. In general, mammals living in environments with stronger UV radiation tend to have darker body colors than relatives living in environments with less UV radiation [27]. In particular, the colors of human skin and irises are believed to be adaptations for protecting tissues from UV radiation, as stronger UV radiation (e.g. in low-latitude or high-altitude area) increases melanin production [27]–[29].

However, from this perceptive, explaining the iris color of gray wolves and its changes with growth is difficult. Iris color did not significantly differ among high arctic wolves, Canadian wolves, and Mexican wolves living in arctic, temperate, and subtropical areas, respectively. In addition, the irises and body coat of gray wolf cubs are darkly pigmented for about 1 month after birth while they remain in the dark den (with less UV) and change to lighter colors after they begin to live outside of the den (with stronger UV). This color change with growth is opposite of what would be predicted from the “UV radiation theory.” In addition, this theory cannot explain why many species with A-type faces have a light-colored coat around the eyes (high CE and/or CF).

In contrast, the relationships of facial color type with sociality and gazing behavior suggest that the A-type faces of canid species are adaptations for visual communication via the gaze signal. The fact that the studied canid species with A-type faces tended to engage in group living suggests that they use the gaze signal in communication among group members, as the need for communication is larger for group-living species than for solo/pair-living species. Group-hunting species had significantly lighter iris color (an important factor of gaze-signaling eyes) than those of solo-hunting species. These results suggest that the gaze signal is used for communication during group hunting in many of these species, although a significant relationship between facial color type and hunting behavior type was not detected.

The longest duration time for gazing behavior was observed in wolves, a species with an A-type face, which also suggests that A-type faces in canid species serve as adaptations for visual communication via the gaze signal. Gazing behavior would facilitate the easy detection of the gaze signal by other conspecifics. Because wolves directed their faces toward others while keeping their entire body still during these behaviors, their gaze signal could be clearly displayed to others even from a distance. Pointing is a behavior characteristic to gray wolves that occurred for a particularly long duration (7.67 s on average). Face-averting behavior was observed in all three focal species, further suggesting the importance of the gaze signal in these species, at least at close proximities. Fox [4] also reported that “in the wolf, coyote and domesticated dog, exaggerated looking away with a marked turning of the head and neck occurs during play.” Together, these results suggest that gray wolves use the gaze signal in communication among group members.

The relationship between facial color type and phylogeny (Figure 4) suggests that A-type faces with light-colored irises evolved independently in the wolf-like clade and the red fox-like clade. In the red fox-like clade, in which 7 of 10 study species were categorized as solo/pair-living, A-type faces were observed in three species, including a group-living and group-hunting species (Corsac foxes) and species with relatively higher sociality within this clade (arctic foxes and red foxes). Arctic foxes have been reported to share carcasses of large prey with other individuals [12], and non-breeding individuals sometimes form temporary groups [30]. In red foxes, the basic social unit is a pair, but groups with up to six members (usually one adult male and 2–5, probably related females) may share a territory, depending on habitat; non-breeding females in the groups may care the cubs as helpers [31].

In the wolf-like clade in which all species engage in group living, 8 of 10 study species exhibit A-type faces (African wild dogs and dingoes do not). African wild dogs with C-type faces are known to wave their white-tipped tails in an upright position like flags during group hunting (images from [32]), suggesting that they use their white tails rather than their facial color pattern as visual signals. In addition, African wild dogs often vocalize various types of sounds, suggesting a developed acoustic communication [33], [34]. Dingoes can also use acoustic signals rather than the gaze signal, as they vocalize three types of howling with 10 variations [35]. Furthermore, bush dogs of the South American clade, the other exceptional species with C-type faces that engages in group living and group hunting, may also primarily use acoustic signals for communication [17]. We observed captive bush dogs frequently vocalizing, even when walking. In contrast, the captive gray wolves made almost no sound even during play or fighting, except occasional distress voices (whimpers) and agonistic voices (growls) when attacked. Therefore, such species with B- or C-type faces that in engage group living may use acoustic and/or other visual signals instead of the gaze signal as their primary means of communication.

The enhanced gaze-signal of the A-type face could be disadvantageous during hunting, as prey animals could use the gaze signal to realize that they have been targeted by the predator and then prepare to escape. Cott [1] pointed out that solo-hunting predators such as frogs and snakes often have face colorations camouflaging their gaze direction (C-type faces). B-type faces with dark irises obscuring the gaze direction may also serve to deceive natural prey animals, making it difficult for prey animals to know if the predator has them in their gaze. This strategy could be one reason why solo/pair-living species tended to have B-type faces, as they are also solo-hunting species.

Many previous studies have demonstrated the importance of visual communication in gray wolves via their use of various visual signals, including facial expressions and expressions of the ear, body, and tail postures [36]–[38]. Although few studies have examined gaze-signal communication in wolves, Range and Virányi [7] recently reported that gray wolves could understand the gaze and head direction of domestic dogs and humans and stare in the same direction. Even 3-months-old gray wolves were reported to understand the gaze direction of humans [7]. In gray wolves, the gazing behavior to humans (about 0.5 s on average) [6] was shorter than that to the conspecifics observed in this study (3.32 s on average). These facts suggest that the gaze signal of gray wolves is mainly used for communication among conspecifics.

Domestic dogs which share a genetic basis for conspecific gaze-communication with wolves can also understand the gaze signal of other dogs and humans [4]–[6]. The duration time of gazing behavior to humans was longer in domestic dogs (about 1.00 s on average) than in gray wolves (about 0.5 s on average) [6]. It suggests that domestic dogs which showed longer gazing behavior to humans regarding as communication partners have been selected artificially. In dogs, however, facial color patterns may have diversified during domestication by artificial selection.

Our comparison of face morphology and gazing behavior among canid species highlighted the gaze-signal communication in species with A-type faces, especially gray wolves, and provided a novel perspective for studies on their communication and morphology.

## Supporting Information

Table S1 **List of studied species and source of the facial images.** The number from SI1 to SI26 before parentheses is the literature number in the reference list of Table S1. The number in parentheses is the number of the studied images.
(XLSX)

Table S2 **Contrast indices of the studied animals.** The rank of each index was shown in the parenthesis. Indices of different body color types in gray wolves (black, gray and white), red foxes (black, red and silver) and arctic foxes (white in winter, grayish-white and black in summer) were shown separately. Asterisks (*) before indices means that the values were larger than the average of each index among studied species.
(XLSX)

Table S3 **Gazing and face-averting behavior of the three group-living species.** Gazing behavior was defined as a behavior during which the animal fixes its face direction toward the other individual for over 1 s, keeping its body still in standing, crouching, sitting, lying down, upside down, or pointing posture. Face-averting behavior was defined as a behavior during which the animal keeps its face averted from other animal in vicinity of that animal for over 1 s.
(XLSX)
